# Beclin 1, LC3 and P62 Expression in Equine Sarcoids

**DOI:** 10.3390/ani12010020

**Published:** 2021-12-23

**Authors:** Manuela Martano, Gennaro Altamura, Karen Power, Pierluigi Liguori, Brunella Restucci, Giuseppe Borzacchiello, Paola Maiolino

**Affiliations:** 1 Department of Veterinary Medicine and Animal Productions, University of Naples Federico II, Via F. Delpino 1, 80137 Naples, Italy; gennaro.altamura@unina.it (G.A.); karen.power@unina.it (K.P.); brunella.restucci@unina.it (B.R.); giuseppe.borzacchiello@unina.it (G.B.); paola.maiolino@unina.it (P.M.); 2 DVM, EQUINE CLINIC Punto Verde, 81110 Caserta, Italy; pierluigi.liguori@hotmail.it

**Keywords:** autophagy, equine sarcoids, BPV

## Abstract

**Simple Summary:**

Equine sarcoids, caused by bovine papillomaviruses, are equine skin tumors of fibroblastic origin. It is well known that bovine papillomaviruses are able to interfere with the survival and proliferation of cells by regulating autophagy, a mechanism implicated in the breakdown and reuse of old and damaged cellular material. The present study focused on the evaluation in equine sarcoids and normal skins of the expression level of some of the main proteins involved in the autophagic pathway, such as Beclin 1, LC3 and P62, by immunohistochemical and biochemical techniques. Results obtained in equine sarcoids suggested an alteration of the autophagic process which could lead to a predominance of a particular population of fibroblast. Those fibroblasts could survive longer in a hypoxic microenvironment and produce more and/or altered collagen, giving an origin to the equine sarcoid.

**Abstract:**

Background: It is well known that δ-bovine papillomaviruses (BPV-1, BPV-2 and BPV-13) are one of the major causative agents of equine sarcoids, the most common equine skin tumors. Different viruses, including papillomaviruses, evolved ingenious strategies to modulate autophagy, a complex process involved in degradation and recycling of old and damaged material. Methods: The aim of this study was to evaluate, by immunohistochemistry (IHC) and Western blot (WB) analysis, the expression of the main related autophagy proteins (Beclin 1, protein light chain 3 (LC3) and P62), in 35 BPV1/2 positive equine sarcoids and 5 BPV negative normal skin samples. Results: Sarcoid samples showed from strong-to-moderate cytoplasmic immunostaining, respectively, for Beclin 1 and P62 in >60% of neoplastic fibroblasts, while LC3 immunostaining was weak to moderate in ≤60% of neoplastic fibroblasts. Western blot analysis confirmed the specificity of the antibodies and revealed no activation of autophagic flux despite Beclin 1 overexpression in sarcoid samples. Conclusion: Results could suggest the activation of the initial phase of autophagy in equine sarcoids, and its impairment during the following steps. The impairment of autophagy could lead to a selection of a quiescent population of fibroblasts, which survive longer in a hypoxic microenvironment and produced more and/or altered collagen.

## 1. Introduction

Normal cell growth requires a balance between the rates of protein synthesis and its degradative processes [1]. The two major quality control pathways responsible for cellular degradation are the ubiquitin–proteasome system and autophagy. Whereas the first is involved in degradation of small, short-lived proteins, autophagy is the preferred degradative process for large, heterogeneous cytoplasmic materials [2,3,4]. Autophagy is a complex process that consists of several sequential steps: (1) initiation; (2) elongation; (3) maturation; (4) degradation [2,5,6]. All these steps are controlled by several proteins, encoded by more than 30 autophagy-related genes, which lead to newly synthesized double-membrane vesicles encapsulating cytosolic material, which is delivered to the lysosomes for proteolytic digestion [4,7]. Beclin 1 protein, encoded by the BECN1 gene, plays a central role in the initiation stage of autophagic vesicle formation that induces the creation of an active phagophore [8,9]. Beclin 1 binds protein light chain 3 (LC3), which is associated with autophagosomal membranes that engulf cytoplasmic content for subsequent degradation. During this process, the cytosolic isoform LC3I is converted to the membrane-bound LC3II form [10] and then binds to the adaptor protein P62 sequestrome (SQSTM1), which is selectively degraded by autophagy [11]. 

Although initially believed to have only a physiological role in the degradation and recycling of old and damaged cytoplasmic material, it is now accepted that autophagy is also closely related to many pathological conditions, such as cardiovascular diseases [12], neurodegeneration [13], cancer [14] and viral infections [15]. It is well known that several viruses evolved different ingenious strategies to modulate autophagy, using them to increase their survival or proliferation [15]. Recently, HPV16 has emerged as an oncogenic virus with the ability of manipulating the autophagic process through the inhibition of the final autophagosome–lysosoma fusion [16]. Moreover, δ-bovine papillomavirus oncoproteins have been shown to interact with a network of proteins involved in the autophagy of urothelial cancer cells infected by BPV1/2 [17,18]. 

Numerous studies have provided evidence for the direct involvement of δ-bovine papillomavirus infection (BPV-1, BPV-2 and BPV-13) in the pathogenesis of equine sarcoids [19,20,21,22]. Equine sarcoids are locally invasive, fibroblastic skin tumors that represent the most common tumor in equids worldwide [23,24]. They are often correlated with a high recurrence rate after surgical excision [25,26,27] and can significantly impact the function or aesthetics of horses leading to a decrease of economic value of affected animals. It was already accepted that infection alone is not sufficient for tumor development, for which the concomitant presence of other factors such as chronic physical trauma, genetical predisposition and alteration of the wound healing process, is necessary [28,29,30]. Although sarcoid is one of the most common pathologies in horses, its pathogenetic mechanism is not completely known and an effective therapy has not yet been defined. 

In our previous studies, we have suggested that, in genetically predisposed equines an altered wound healing process could create a microenvironment that activates BPV latent infection, leading to neoplastic transformation and sarcoid development [31,32,33]. Altered turnover of the extracellular matrix (ECM) deposition and degradation is reported as the basic mechanism for these changes [31] and is strictly correlated to angiogenesis induced in response to hypoxia [32,33]. 

There is growing evidence that autophagy could have a dual role in tumor development, as a tumor suppressor by inhibiting cancer cell survival, but also as a tumor promoter by increasing the ability of cancer cells to live in a nutrient starvation and hypoxic environment [34].

In this regard, the present study focused on the evaluation, by immunohistochemical and biochemical analysis, of the expression level of some of the main autophagy-related proteins, such as the Beclin 1, LC3 and P62 in BPV1/2 positive equine sarcoids and BPV negative normal skins. 

## 2. Materials and Methods

### 2.1. Tumor Samples

A total of 35 equine sarcoids were sampled from clinically affected horses following the Directive 2010/63/EU (art. 1). Samples originated from lesions localized on the neck (*n* = 2), abdomen (*n* = 6), head (*n* = 6), limbs (*n* = 10), chest (*n* = 8), and paragenital region (*n* = 3) (Table 1). Moreover, 5 skin samples, were collected during necropsy from the neck (*n* = 1), abdomen (*n* = 1), head (*n* = 1), limb (*n* = 1) and chest (*n* = 1) of healthy horses.

Samples were routinely processed for histopathological examination. Briefly, they were 10% formalin-fixed, paraffin-embedded and stained with haematoxylin and eosin (HE). For 5 out of the 35 sarcoid samples mentioned above, half of the biopsy was frozen at −80 °C immediately after collection, along with 3 additional normal skin samples, to then be analyzed by Western blot. 

All sarcoid samples, the same as those previously used [31,32,33], were BPV1/2 positive, while normal skin samples were BPV negative [35]. 

### 2.2. Immunohistochemistry 

Immunohistochemistry was performed on 5 μm sections using the streptavidin–biotin–peroxidase method. After deparaffinization in alcohol decreasing solutions, sections were incubated in 0.3% H_2_O_2_ in methanol for 20 min to block endogenous peroxidase activity. Antigen retrieval was obtained by microwave heating (twice for 5 min each at 750 W) in citrate buffer, pH 6.0. The slides were then washed three times with phosphate buffered saline (PBS, pH 7.4, 0.01 M), and incubated with normal goat serum (Santa Cruz Biotechnology, CA, USA) diluted at 20% in PBS for 1 h at room temperature (rt). The following primary antibodies were applied overnight at 4 °C: polyclonal rabbit anti-human Beclin 1 antibody (H300: sc-11,427, Santa Cruz Biotechnology, Dallas, TX, USA) (predicted to cross-react with horse) diluted 1:200 in PBS; polyclonal rabbit anti-human LC3A/B antibody (ab128025; abcam) (predicted to cross-react with horse) diluted 1:100 in PBS; polyclonal rabbit anti-human SQSTM1 /P62 antibody (ab101266; abcam) (predicted to cross-react with horse) diluted 1:1000 in PBS.

Control sections (equine normal skin and sarcoid) were incubated with PBS and with rabbit IgG (purified rabbit IgG P120-201-Bethyl Laboratories, Inc.) instead of the primary antibody using the same concentration as the primary antibodies. Finally, sections were counterstained with haematoxylin, and the immunoreaction manifested after DAB (diaminobenzidine tetrahydrochloride) application. 

### 2.3. Scoring of Immunoreactivity 

To evaluate the expression of Beclin 1, two independent observers (Martano M. and Maiolino P.) under blinded conditions applied a semiquantitative score according to the following technique: the number of immunoreactive cells was evaluated by counting 1000 cells in 10 fields at 400× magnification (40× objective 10× ocular), and the results were formulated as percentages and scored as follows: 0 (≤10% positive cells); 1 (10–40% positive cells); 2 (40–60% positive cells); and 3 (>60% positive cells). Moreover, the intensity of immunostaining was labeled as n.a. (not assessable), − (negative staining), +/− (weak immunostaining), + (moderate immunostaining), and ++ (strong immunostaining) (Table 1), as in a previous study [32,33].

### 2.4. Western Blot (WB)

Tissue samples were minced and subjected to homogenization, protein extraction, sodium–dodecyl–sulfate polycrylamide gel electrophoresis and WB as described in a previous work [31]. Hela whole-cell lysate was run as a positive control as suggested by the antibodies’ datasheets. Saos-2 cells were employed as a further positive control for Beclin 1 [36]. Background blocking was obtained by incubating the nitrocellulose membranes with Tris buffered saline (TBS: 10 mM Tris-HCl, pH 7.4, 165 mM NaCl)/Tween 0.1% (TBST) supplemented with bovine serum albumin (BSA) at 5% for 1 h at rt, then the anti-Beclin 1, anti-P62 and anti-LC3 antibodies at 1:500 dilution in TBST/BSA 5% were applied over night at 4 °C. After washing steps in TBST, membranes were incubated with donkey anti-rabbit secondary antibody (Bethyl Laboratories #A-120-108P) diluted at 1:2000 in TBST/BSA 5%, then washed and incubated with enhanced luminol-based chemiluminescent substrate (Clarity Western ECL, Bio-Rad Laboratories, Milano, Italy). Each membrane was then stripped and incubated with β-actin antibody at 1:500 dilution (CP01, Calbiochem, San Diego, CA, USA) to allow normalization. Protein bands visualization, densitometric analysis and normalization were obtained by using the Image Lab software run on the ChemiDoc Gel Scanner (Bio-Rad Laboratories, Milano, Italy). 

### 2.5. Statistical Analysis

Statistical analysis was performed applying Student’s *t*-test by using SPSS 17.0 software (SPSS Inc., Chicago, IL, USA). The differences were considered statistically significant for * *p*< 0.05.

## 3. Results

### 3.1. Histological and Immunohistochemical Results

All sarcoid samples showed the typical histological features, as previously reported [28,31,32,33].

Results from immunohistochemical analysis were reported in Table 1. All samples showed cytoplasmic immunostaining of macrophages, which have been used as internal positive controls. 

All normal skin samples (5/5) showed a weak cytoplasmic positivity (score +/−) for Beclin 1 and LC3 in 40–60% of cells located in the basal epidermis (score 2), while fibroblasts were negative. Moderate nuclear (score +) and weak cytoplasmic (score +/−) expression of P62 were predominantly detected in 40–60% of cells located in the stratum spinosum of epidermis (score 2), while fibroblasts were negative.

**Figure 1:**
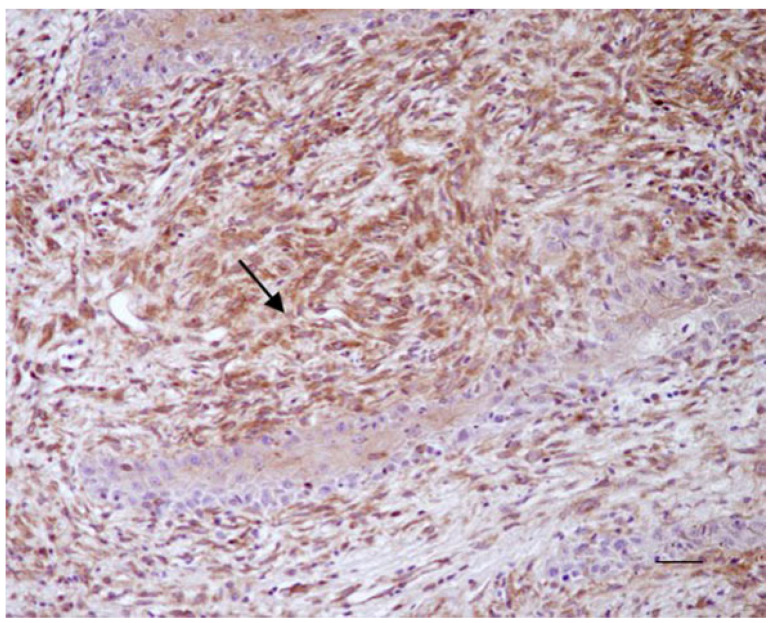
Equine sarcoid. Immunohistochemical staining. Neoplastic fibroblasts showed strong brown immunostaining for Beclin 1 (arrow) (20× Scale bar: 100 μm).

**Figure 2:**
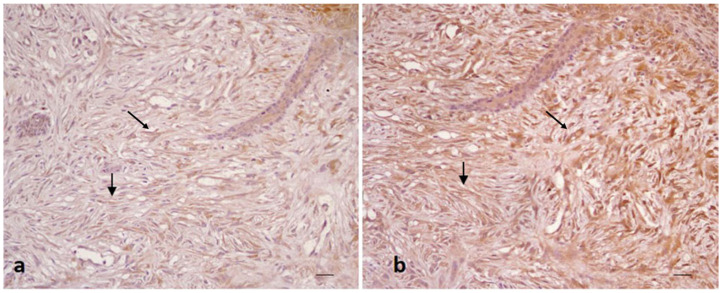
Equine sarcoid. Immunohistochemical staining. Neoplastic fibroblasts with moderate (**a**) and strong (**b**) positivity for LC3 (**a**) and P62 (**b**) (20× scale bar: 100 μm).

All sarcoid samples (35/35; 100%) showed positivity for Beclin 1 in >60% of neoplastic fibroblasts (score 3). The immunostaining was granular and cytoplasmic and resulted moderate (+) in 10/35 samples (29%) and strong (++) in 25/35 (71%) samples (Figure 1). For LC3, 22/35 sarcoid samples (63%) showed weak positivity (+/−) in 10–40% of neoplastic fibroblasts (score 1), while 13/35 sarcoid samples (37%) showed a moderate immunostaing (+) in 40–60% of neoplastic fibroblasts (score 2) (Figure 2a). 

All sarcoid samples (35/35; 100%) showed strong immunostaing for P62 (++) in >60% of neoplastic cells (score 3) (Figure 2b).

### 3.2. Biochemical Results 

**Figure 3:**
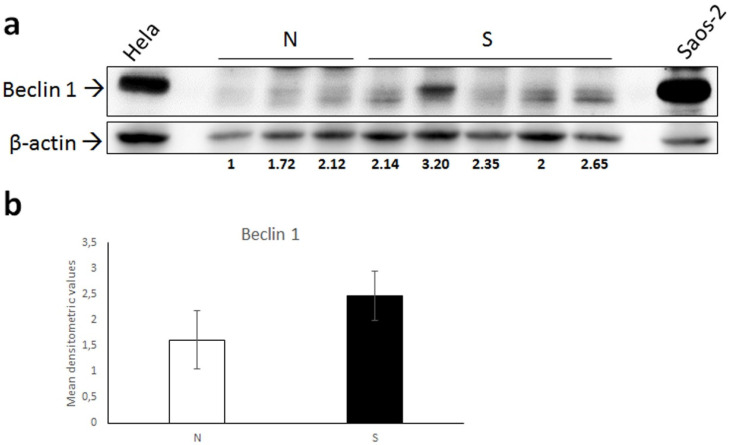
(**a**) Western blot analysis for Beclin 1 in normal skin samples (N) and equine sarcoids (S). Cell lysates from Hela and Saos-2 cell lines were run on the gel to demonstrate the specificity of the antibody. Following stripping of the membrane, anti β-actin antibody was applied in order to allow normalization. Densitometric ratio Beclin 1/β-actin for each normal and sarcoid sample is indicated. (**b**) Mean densitometric values +/− standard deviation of N vs. S group (original Western blot figure is in Figure S1).

WB analysis for Beclin 1 showed detection of a band of the predicted molecular weight (~52 kDa) in all samples, Hela and Saos-2 cell lysate used as a positive control, demonstrated the specificity of the antibody [36] (Figure 3a). The intensity of the Beclin 1 bands was higher in four out of five tumors; however, the difference recorded between normal skin and sarcoid samples was not statistically significant (Figure 3b). 

**Figure 4:**
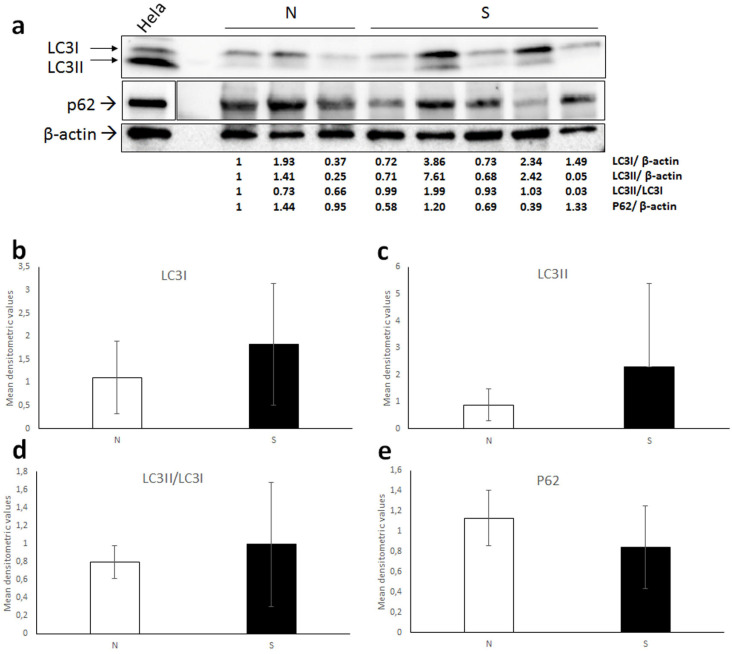
(**a**) Western blot analysis for LC3 and P62 in normal skin samples (N) and equine sarcoids. Cell lysate from Hela cell line was run on the gel to demonstrate the specificity of the antibodies. For P62 blot, boxes are cut from the same membrane at different exposure times and properly aligned according to the molecular weight loaded onto the gel (see Figure S2). Following stripping of the membrane, anti β-actin antibody was applied in order to allow normalization. Densitometric ratios LC3I/ β-actin, LC3II/ β-actin, LC3II/ LC3I and P62/ β-actin for each normal and sarcoid sample are indicated. (**b**–**e**) Mean densitometric values +/− standard deviations of N vs. S group (original Western blot figure is in Figure S2).

When performing WB for LC3, the antibody recognized a double band in equine lysates, and as expected, in Hela whole-cell extract, corresponding to LC3I (16 kDa) and LC3II (14 kDa) (Figure 4a). Quantification of bands by densitometric analysis showed high variability of both LC3I and LC3II expression levels, indeed no significant difference between normal and sarcoid tissues was revealed by *t*-test for neither of the two isoforms (Figure 4b,c). Additionally, when calculating the densitometric ratio LC3II/LC3I as a measure of activated autophagy, mean values of normal vs. sarcoid groups were comparable and no statistically significant difference was found (Figure 4d).

WB for P62 in equine samples and Hela cells confirmed the specificity of the antibody by detecting a band of the expected molecular mass (~62 kDa) (Figure 4a); however, densitometric measurement followed by statistical analysis (*t*-test) revealed highly variable expression levels and no significant difference between normal cell and sarcoid lysates (Figure 4e). 

## 4. Discussion

Beclin 1, LC3 and P62 are the key mediators of autophagy and are widely used as markers for monitoring and reliably quantifying autophagic activities in normal and pathological conditions [34]. Beclin 1 is essential for the activation of autophagic process, as it interacts with a variety of cofactors to trigger the autophagy protein cascade; LC3 is widely used as an effective indicator of autophagic flux, as, by binding to P62, it regulates membrane elongation and autophagosome maturation [4,34]. Moreover P62, is involved in the transport of altered proteins to degradation and is in turn degraded by autophagy [37,38].

Dysfunctions of these autophagy-related proteins have been implicated in many disorders, including human cancers, in which both reduced expression and overexpression may occur [39,40,41,42,43,44,45,46]. 

This variable expression in different types of cancers could be linked to the specificity of autophagic activity in different organs and histologic types, and to the different role of autophagy that may either induce or inhibit tumor cell survival. On the one hand, they may play a role as tumor promoters, by enhancing stress tolerance and supplying nutrients to meet the metabolic demand of neoplastic cells. On the other hand, they may act as a mechanism of tumor suppression, limiting carcinogenesis. The tumor suppression function is mediated by the reduction in damaged organelles and proteins and the prevention of DNA damage, leading to the maintaining of cellular homeostasis and survival, in an energy-deficient environment [14,34].

To our knowledge, only few studies have focused on autophagy in spontaneous animal cancers [47,48]. 

In this study we assessed, for the first time, Beclin 1, LC3 and P62 expression levels in a series of BPV 1 and BPV 2 positive equine sarcoids by immunohistochemistry and biochemical analysis.

All normal skin samples showed a weak/moderate immunostaining for Beclin 1, LC3 and P62 in epidermal cells, where they are known to be constitutively expressed [49].

Sarcoid samples showed moderately or strongly immunostaining for Beclin 1 in most neoplastic fibroblasts (>60%). The overexpression of Beclin 1 highlighted in this study could be related to a condition of hypoxic microenvironment that happens in sarcoids in consequence of the excessive and progressive deposition of collagen and of the presence of vessels that are not able to perfectly oxygenate the tissue [31,32,33]. It is well known that hypoxia induces an increase in Beclin 1 expression and the consequent activation of the autophagic process [50,51,52]. 

Consistently, WB analysis confirmed overexpression of Beclin 1 in most of sarcoid samples, however possible concerns due to the small number of samples analyzed should be taken into account.

Furthermore, we found a weak/moderate staining for LC3 and a strong immunostaining for P62 in sarcoid samples, by immunohistochemistry. Consistently, despite in a limited number of samples, WB of LC3 and P62 confirmed no occurrence of autophagy, as no significant differences in their expression or activation could be detected between tumor vs. normal skin group.

Taken together, these results suggest that in fibroblasts of equine sarcoids there could be an impairment of autophagic processes, during the phase of membrane elongation and autophagosome maturation. Therefore, it is plausible that the activation of the initial phase of autophagy, evidenced through the overexpression of Beclin 1, may not be followed by the next steps, resulting in defective or impaired autophagic capability of neoplastic fibroblasts. It is reported that a defective autophagy could contribute to the production of excess or altered extracellular matrix protein such as collagen [53,54,55]. Thus, it would seem plausible that the more collagen is synthesized (e.g., in tissues with a high turnover) the higher will be the chance of abnormal molecules being formed [55]. These results seem to confirm our previous study in which we have indeed shown that the excessive and progressive deposition of connective tissue (collagen) in sarcoids might not only be the result of a deficiency in matrix degradation, but also caused by an elevated synthesis of collagen by neoplastic fibroblasts [31]. Furthermore, the activation of the initiation phase of autophagy by Beclin 1 could lead to the recycling of essential nutrients for the supply of energy, guaranteeing neoplastic fibroblasts survival, in a hypoxic microenvironment [51]. In conclusion, it is possible that a quiescent population of sarcoid fibroblasts, which survived longer and produced more and/or altered collagen due to a defective or impaired autophagic capability, is selected.

In the present study we have evaluated the expression of autophagic markers in BPV1 and BPV2 positive equine sarcoids, compared to BPV negative normal skin controls. Most of the oncogenic viruses possess the capability to inhibit autophagy mainly promoting viral replication and cellular maintenance, though this favors cancer onset [15,16]. There are many conflicting results about the role of papillomavirus (PVs) in autophagy leading to carcinogenesis [56,57]. HPV blocks autophagy, through the inactivation of the mRNA expression of autophagic genes, leading to down-regulation of phagophore assembly [16]. However, only a small number of studies correlate the reduction in autophagy with cancer development in HPV pseudo latent infections [16]. Although we did not demonstrate the direct interaction between autophagy-related proteins and BPV1/2, we cannot exclude that viral oncoproteins could be responsible for the down-regulation or impairment of the autophagic process in equine sarcoids. 

Currently, despite the numerous treatment options available for equine sarcoids, not all of them are totally effective, with consequent frequent recurrences. This study paves the way for additional work in vitro, which may contribute to a better understanding of autophagic processes in equine sarcoids and the possible role of BPV1/2 in its regulation, representing a step forward in the unveiling of its pathogenesis and ideation of new possible therapeutic strategies. 

## Figures and Tables

**Table 1:** Staining intensity and percentage positive scores for Beclin 1, LC3 and P62 in 35 BPV positive equine sarcoids.

Location	Number of Cases	Staining Intensity Score * Beclin 1	Percentage Positive Score ** Beclin 1	Staining Intensity Score * LC3	Percentage Positive Score ** LC3	Staining Intensity Score * P62	Percentage Positive Score ** P62
Neck	2	++	3	+/−	1	++	3
Limb	6	++	3	+/−	1	++	3
	4	+	3	+/−	1	++	3
Abdomen	4	++	3	+/−	1	++	3
	2	+	3	+	2	++	3
Pectoral region	5	++	3	+	2	++	3
	3	+	3	+	2	++	3
Head	5	++	3	+/−	1	++	3
	1	+	3	+/−	1	++	3
(para)-genital region	3	++	3	+	2	++	3

* Staining intensity score: − negative staining; +/− weak immunolabelling; + moderate immunolabelling; ++ strong immunolabelling; ** per-centage positive score: 0 (≤10% positive cells); 1 (10–40% positive cells); 2 (40–60% positive cells); 3 (>60% positive cells).

## Data Availability

Not applicable.

## Supplementary Materials

The following are available online at https://www.mdpi.com/article/10.3390/ani12010020/s1. The data sets supporting the results of this article are included within the article and its additional files. Figure S1: Original Western Blot figure of Figure 3. Figure S2: Original Western Blot figures of Figure 4.

## List of Abbreviations

BPV: bovine papillomavirus; HPVs: human papillomaviruses; HIF1: hypoxia-inducible factor-1 alpha (HIF-1α); VEGF: vascular endothelial growth factor; IHC: immunohistochemistry; WB: Western blot; N: normal skin; ECM: extracellular matrix; LC3: protein light chain 3; SQSTM1: sequestosome-1.

## References

[1] Rothman S. (2010). How is the balance between protein synthesis and degradation achieved?. Theor. Biol. Med. Model..

[2] Klionsky D.J., Emr S.D. (2000). Autophagy as a regulated pathway of cellular degradation. Science.

[3] Kroemer G. (2015). Autophagy: A druggable process that is deregulated in aging and human disease. J. Clin. Investig..

[4] Dikic I. (2017). Proteasomal and autophagic degradation systems. Ann. Rev. Biochem..

[5] Parzych K.R., Klionsky D.J. (2014). An Overview of Autophagy: Morphology, Mechanism, and Regulation. Antioxid. Redox Signal..

[6] Tooze S.A., Schiavo G. (2008). Liaisons dangereuses: Autophagy, neuronal survival and neurodegeneration. Curr. Opin. Neurobiol..

[7] Münz C. (2009). Enhancing immunity through autophagy. Annu. Rev. Immunol..

[8] Tukaj C. (2013). The significance of macroautophagy in health and disease. Folia Morphol..

[9] Itakura E., Kishi C., Inoue K., Mizushima N. (2008). Beclin 1 forms two distinct phosphatidylinositol 3-kinase complexes with mammalian Atg14 and UVRAG. Mol. Biol. Cell.

[10] Kabeya Y., Mizushima N., Ueno T., Yamamoto A., Kirisako T., Noda T., Kominami E., Ohsumi Y., Yoshimori T. (2000). LC3, a mammalian homologue of yeast Apg8p, is localized in autophagosome membranes after processing. EMBO J..

[11] Pankiv S., Clausen T.H., Lamark T., Brech A., Bruun J.A., Outzen H., Øvervatn A., Bjørkøy G., Johansen T. (2007). p62/SQSTM1 binds directly to Atg8/LC3 to facilitate degradation of ubiquitinated protein aggregates by autophagy. J. Biol. Chem..

[12] Lavandero S., Chiong M., Rothermel B.A., Hill J.A. (2015). Autophagy in cardiovascular biology. J. Clin. Investig..

[13] Frake R.A., Ricketts T., Menzies F.M., Rubinsztein D.C. (2015). Autophagy and neurodegeneration. J. Clin. Investig..

[14] White E. (2015). The role for autophagy in cancer. J. Clin. Investig..

[15] Jackson W.T. (2015). Viruses and the autophagy pathway. Virology.

[16] Mattoscio D., Medda A., Chiocca S. (2018). Human papilloma virus and autophagy. Int. J. Mol. Sci..

[17] Roperto S., Russo V., De Falco F., Urraro C., Maiolino P., Del Piero F., Roperto F. (2019). Bovine papillomavirus E5 oncoprotein expression and its association with an interactor network in aggresome-autophagy pathway. Vet. Microbiol..

[18] Roperto S., Russo V., Rosati A., Ceccarelli D.M., Munday J.S., Turco M.C., Roperto F. (2018). Chaperone-assisted selective autophagy in healthy and papillomavirus-associated neoplastic urothelium of cattle. Vet. Microbiol..

[19] Borzacchiello G., Roperto F. (2008). Bovine papillomaviruses, papillomas and cancer in cattle. Vet. Res..

[20] Lunardi M., Alcântara B., Otonel R., Rodrigues W., Alfieri A., Alfieri A. (2013). Bovine papillomavirus type 13 DNA in equine sarcoids. J. Clin. Microbiol..

[21] Chambers G., Ellsmore V.A., O’Brien P.M., Reid S.W., Love S., Campo M.S., Nasir L. (2003). Association of bovine papillomavirus with the equine sarcoid. J. Gen. Virol..

[22] Bogaert L., Martens A., Kast W.M., Van Marck E., De Cock H. (2010). Bovine papillomavirus DNA can be detected in keratinocytesof equine sarcoid tumors. Vet. Microbiol..

[23] Marti E., Lazary S., Antczak D.F., Gerber H. (1993). Report of the first international workshop on equine sarcoid. Equine Vet. J..

[24] Ragland W.L., Keown G.H., Spencer G.R. (1970). Equine sarcoid. Equine Vet. J..

[25] Borzacchiello G., Corteggio A. (2009). Equine sarcoid: State of the art. Ippologia.

[26] Nasir L., Campo M.S. (2008). Bovine papillomaviruses: Their role in the aetiology of cutaneous tumors of bovids and equids. Vet. Dermatol..

[27] Nasir L., Brandt S. (2013). Papillomavirus associated diseases of the horse. Vet. Microbiol..

[28] Knottenbelt D.C. (2005). A suggested clinical classification of the equine sarcoid. Clinical techniques in equine. Practice.

[29] Cochrane A.C. (1997). Models in vivo of wound healing in the horse and the role of growth factors. Vet. Dermatol..

[30] Hanson R.R. (2008). Review: Complications of equine wound management and dermatologic surgery. Vet. Clin. N. Am. Equine Pract..

[31] Martano M., Corteggio A., Restucci B., De Biase M.E., Borzacchiello G., Maiolino P. (2016). Extracellular matrix remodeling in equine sarcoid: An immunohistochemical and molecular study. BMC Vet. Res..

[32] Martano M., Power K., Restucci B., Pagano I., Altamura G., Borzacchiello G., Maiolino P. (2018). Expression of vascular endothelial growth factor (VEGF) in equine sarcoid. BMC Vet. Res..

[33] Martano M., Altamura G., Power K., Restucci B., Carella F., Borzacchiello G., Maiolino P. (2020). Evaluation of Hypoxia-Inducible Factor-1 Alpha (HIF-1α) in Equine Sarcoid: An Immunohistochemical and Biochemical Study. Pathogens.

[34] Yun C.W., Lee S.H. (2018). The Roles of Autophagy in Cancer. Int. J. Mol. Sci..

[35] Borzacchiello G., Russo V., Della Salda L., Roperto S., Roperto F. (2008). Expression of platelet-derived growth factor-beta receptor and bovine papillomavirus E5 and E7 oncoproteins in equine sarcoid. J. Comp. Pathol..

[36] He J.M., Liu P.Y., Wang J. (2020). MicroRNA-17-5p regulates the growth, migration and invasion of the human osteosarcoma cells by modulating the expression of PTEN. J. BUON.

[37] Katsuragi Y., Ichimura Y., Komatsu M. (2015). p62/SQSTM1 functions as a signaling hub and an autophagy adaptor. FEBS J..

[38] Liu J.-L., Chen F.-F., Lung J., Lo C.-H., Lee F.-H., Lu Y.-C., Hung C.-H. (2014). Prognostic significance of p62/SQSTM1 subcellular localization and LC3B in oral squamous cell carcinoma. Br. J. Cancer.

[39] Vega-Rubín-de-Celis S. (2020). The Role of Beclin 1-Dependent Autophagy in Cancer. Biology.

[40] Won K.Y., Kim G.Y., Kim Y.W., Song J.Y., Lim S.J. (2010). Clinicopathologic correlation of beclin-1 and bcl-2 expression in human breast cancer. Hum. Pathol..

[41] Jiang Z.F., Shao L.J., Wang W.M., Yan X.B., Liu R.Y. (2012). Decreased expression of Beclin-1 and LC3 in human lung cancer. Mol. Biol. Rep..

[42] Won K.Y., Kim G.Y., Lim S.J., Kim Y.W. (2012). Decreased Beclin-1 expression is correlated with the growth of the primary tumor in patients with squamous cell carcinoma and adenocarcinoma of the lung. Hum. Pathol..

[43] Ding Z.B., Shi Y.H., Zhou J., Qiu S.J., Xu Y., Dai Z., Shi G.M., Wang X.Y., Ke A.W., Wu B. (2008). Association of autophagy defect with a malignant phenotype and poor prognosis of hepatocellular carcinoma. Cancer Res..

[44] Miracco C., Cevenini G., Franchi A., Luzi P., Cosci E., Mourmouras V., Monciatti I., Mannucci S., Biagioli M., Toscano M. (2010). Beclin 1 and LC3 autophagic gene expression in cutaneous melanocytic lesions. Hum. Pathol..

[45] Ahn C.H., Jeong E., Lee J., Kim M., Kim S., Kim S., Yoo N., Lee S. (2007). Expression of beclin-1, an autophagy-related protein, in gastric and colorectal cancers. Acta Pathol. Microbiol. Immunol. Scand..

[46] Kim H.S., Lee S.H., Do S.I., Lim S.J., Park Y.K., Kim Y.W. (2011). Clinicopathologic correlation of beclin-1 expression in pancreatic ductal adenocarcinoma. Pathol. Res. Pract..

[47] Uchida K. (2017). Pathologic Changes and Autophagy: New Insights for the Pathogenesis of Animal Diseases. Vet. Pathol..

[48] Liu J.L., Chang K.C., Lo C.C., Chu P.Y., Lu C.H. (2013). Expression of autophagy-related protein beclin-1 in malignant canine mammary tumors. BMC Vet. Res..

[49] Akinduro O., Sully K., Patel A., Robinson D.J., Chik A., McPhail G., Braun K.M., Philpott M.P., Harwood C.A., Byrne C. (2016). Constitutive Autophagy and Nucleophagy during Epidermal Differentiation. J. Investig. Dermatol..

[50] Lee S.J., Kim H.P., Jin Y., Choi A.M.K., Ryter S.W. (2011). Beclin 1 deficiency is associated with increased hypoxia-induced angiogenesis. Autophagy.

[51] Tan Q., Wang M., Yu M., Zhang J., Bristow R.G., Hill R.P., Tannock I.F. (2016). Role of Autophagy as a Survival Mechanism for Hypoxic Cells in Tumors. Neoplasia.

[52] Chen Y., Lu Y., Lu C., Zhang L. (2009). Beclin-1 expression is a predictor of clinical outcome in patients with esophageal squamous cell carcinoma and correlated to hypoxia-inducible factor (HIF)-1alpha expression. Pathol. Oncol. Res..

[53] Ranaghan M.J., Durney M.A., Mesleh M.F., McCarren P.R., Garvie C.W., Daniels D.S., Carey K.L., Skepner A.P., Levine B., Perez J.R. (2017). The Autophagy-Related Beclin-1 Protein Requires the Coiled-Coil and BARA Domains to Form a Homodimer with Submicromolar Affinity. Biochemistry.

[54] Del Principe D., Vona R., Giordani L., Straface E., Giammarioli A.M. (2011). Defective autophagy in fibroblasts may contribute to fibrogenesis in autoimmune processes. Curr. Pharm. Des..

[55] Martinez-Outschoorn U.E., Trimmer C., Lin Z., Whitaker-Menezes D., Chiavarina B., Zhou J., Wang C., Pavlides S., Martinez-Cantarin M.P., Capozza F. (2010). Autophagy in cancer associated fibroblasts promotes tumor cell survival: Role of hypoxia, HIF1 induction and NFκB activation in the tumor stromal microenvironment. Cell Cycle.

[56] Everts V., van der Zee E., Creemers L., Beertsen W. (1996). Phagocytosis and intracellular digestion of collagen, its role in turnover and remodelling. Histochem. J..

[57] Wang Z.H., Xu L., Wang Y., Cao M.Q., Li L., Bai T. (2011). Clinicopathologic correlations between human papillomavirus 16 infection and Beclin 1 expression in human cervical cancer. Int. J. Gynecol. Pathol..

