# Supplementary material for: Beclin 1, LC3 and P62 Expression in Equine Sarcoids
Source: Animals (Basel). 2021 Dec 23;12(1):20. doi: 10.3390/ani12010020 (PMC8749869; doi:10.3390/ani12010020)
Supplement: Supplementary file 1 [file animals-12-00020-s001.zip › animals-1479020-supplementary.pdf]

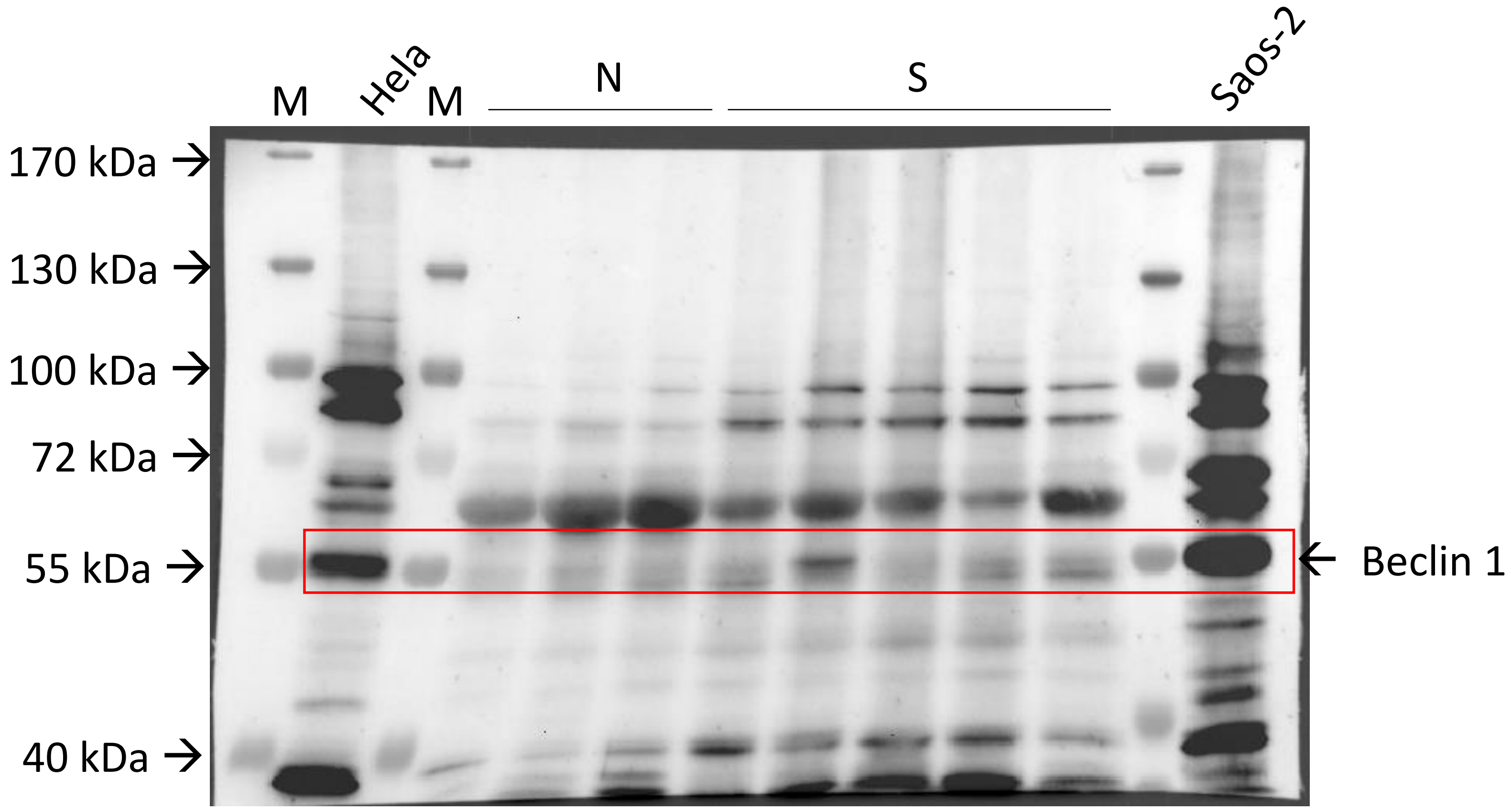

Figure S1. Whole membrane of Western blotting for Beclin 1 from Figure 3, demonstrating detection of a band at the expected molecular size (~52 kDa) in equine tissue samples (N, S), Hela and Saos-2 cell lines. Molecular markers (M) are shown, molecular weights in kDa are indicated on the left of each M band.

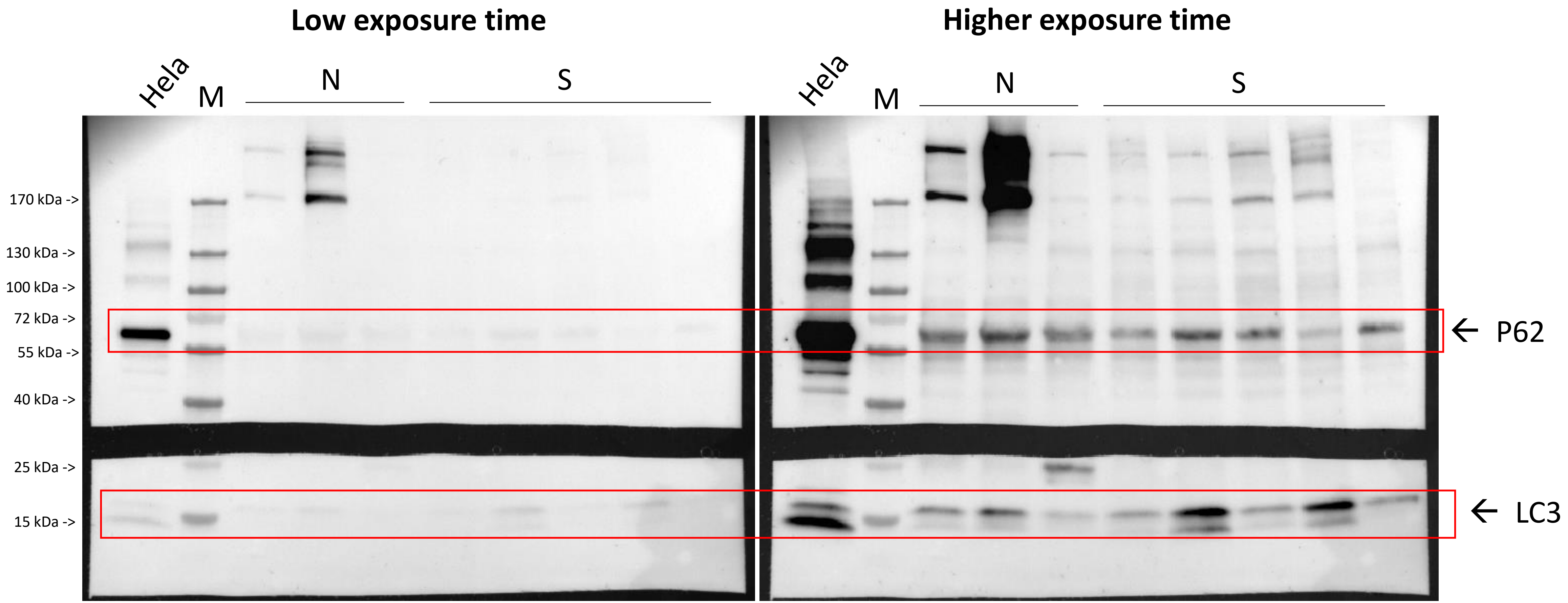

Figure S2. Whole membrane of Western blotting for P62 (upper blot) and LC3 (lower blot) from Figure 4, demonstrating detection of bands at the expected molecular sizes in equine tissue samples (N, S) and Hela cell line. Molecular markers (M) are shown, molecular weights in kDa are indicated on the left of the blots. On the left, the membrane is shown at a low exposure time: at these conditions, P62 band was precisely evaluable in Hela lysate, whilst P62 bands in equine samples as well as LC3 bands in Hela and equine samples were still faint. On the right, the same membrane is shown at higher exposure time: at these conditions, P62 bands were visible also in equine samples and LC3 bands became clearly detectable in Hela along with equine samples.
